# Vestibular disease in dogs under UK primary veterinary care: Epidemiology and clinical management

**DOI:** 10.1111/jvim.15869

**Published:** 2020-08-10

**Authors:** Sinziana Maria Radulescu, Karen Humm, Louis Mark Eramanis, Holger A. Volk, David B. Church, David Brodbelt, Dan Gerard O'Neill

**Affiliations:** ^1^ Department of Clinical Science and Services The Royal Veterinary College North Mymms Herts UK; ^2^ Department of Veterinary Clinical Sciences, Faculty of Veterinary and Agricultural Sciences University of Melbourne Werribee Victoria Australia; ^3^ Department of Small Animal Medicine and Surgery University of Veterinary Medicine Hannover Hannover Germany; ^4^ Department of Pathobiology and Population Sciences The Royal Veterinary College North Mymms Herts United Kingdom

**Keywords:** age related, neurological, stroke, VetCompass

## Abstract

**Background:**

Vestibular disease (VD), central or peripheral, can be a dramatic primary‐care presentation. Current literature describes mostly dogs examined in referral centers.

**Hypothesis/Objectives:**

Describe the prevalence, presentation, clinical management, and outcomes of VD in dogs under primary veterinary care at UK practices participating in VetCompass.

**Animals:**

Seven hundred and fifty‐nine vestibular cases identified out of 905 544 study dogs.

**Methods:**

Retrospective cohort study. Potential VD cases clinically examined during 2016 were verified by reviewing clinical records for signalment, presenting clinical signs, treatments, and outcomes. Multivariable logistic regression was used to evaluate factors associated with VD.

**Results:**

The overall prevalence of VD was 8 per 10 000 dogs (95% CI = 7‐9). Median age at first diagnosis was 12.68 years (interquartile range [IQR], 11.28‐14.64). Compared with crossbreeds, breeds with the highest odds of VD diagnosis included French Bulldogs (odds ratio [OR] = 9.25, 95% CI = 4.81‐17.76, *P* < .001), Bulldogs (OR = 6.53, 95% CI = 2.66‐16.15, *P* < .001), King Charles Spaniels (OR = 4.96, 95% CI = 2.52‐9.78, *P* < .001), Cavalier King Charles Spaniels (OR = 3.56, 95% CI = 2.50‐5.06, *P* < .001), and Springer Spaniels (OR = 3.37, 95% CI = 2.52‐4.52, *P* < .001). The most common presenting signs were head tilt (69.8%), nystagmus (68.1%), and ataxia (64.5%). The most frequently used treatments were antiemetics (43.2%), systemic glucocorticoids (33.1%), antimicrobials (25%), and propentofylline (23.25%). There were 3.6% of cases referred. Improvement was recorded in 41.8% cases after a median of 4 days (IQR, 2‐10.25).

**Conclusions:**

Our study identifies strong breed predispositions for VD. The low referral rates suggest that primary‐care data sources offer more generalizable information for benchmarking to help clinicians review their own clinical activities.

**Abbreviations**
CKCS: Cavalier King Charles Spaniel
CN: cranial nerve
EPR: electronic patient record
IMHA: immune mediated hemolytic anemia
IPVD: idiopathic peripheral vestibular disease
IQR: interquartile range
MUO: meningoencephalitis of unknown origin
OR: odds ratio
VD: vestibular disease

## 1 INTRODUCTION

Vestibular disease (VD) is characterized by a dysfunction of the parts of the nervous system responsible for the maintenance of equilibrium and balance.^1^ The diagnosis of VD is based on cardinal neurological examination abnormalities such as ataxia, head tilt, and pathological nystagmus and strabismus. These characteristic clinical signs can be caused by either central or peripheral neurological dysfunction.^2^ Neurological signs associated with central involvement include proprioception deficits, altered mentation, cranial nerve (CN) deficits other than CN VII or VIII, and vertical or dysconjugate nystagmus. Recognition of these clinical signs can help the clinician differentiate between central and peripheral variants and has an important role in decision making regarding clinical management and when considering referral for advanced imaging. The most common diagnoses in dogs presenting with peripheral VD are otitis media/interna and idiopathic peripheral VD (IPVD).^2^ IPVD is reportedly more common in older dogs and the diagnosis is made by exclusion.^1^ For central VD, multiple causes are reported, including anomalous, metabolic, neoplastic, infectious/inflammatory, traumatic, toxic, and vascular etiologies.^3^

Regardless of the etiology, VD can be a serious welfare concern for affected dogs. Clinical signs are often dramatic on presentation,^4^ with severe disorientation causing distress for both the affected animal and the owner. Although vestibular disorders in dogs are reportedly common,^5^, ^6^, ^7^ there is limited information on the prevalence, risk factors, progression, and outcome of VD in the primary‐care population of dogs because the current veterinary literature is based mainly on the subset of cases presenting to referral institutes. These referral cases are likely to be more complex cases with more financially committed owners and therefore to generalize poorly to the wider population.^8^

The current study aimed to describe the signalment, prevalence, and risk factors in dogs diagnosed with VD in primary‐care practice in the United Kingdom and to describe disease progression, clinical management, and outcomes of these cases.

## 2 MATERIALS AND METHODS

The VetCompass Programme collates de‐identified electronic patient record (EPR) data from primary‐care veterinary practices in the United Kingdom for epidemiological research.^9^ VetCompass collects information fields that include species, breed, date of birth, sex, neuter status and bodyweight, and clinical information from free‐form text clinical notes and summary diagnosis terms (VeNom codes^10^) plus treatment and deceased status with relevant dates. The EPR data were extracted from practice management systems using integrated clinical queries and uploaded to a secure VetCompass structured query language database.^11^

A retrospective cohort study design of dogs attending VetCompass practices was used to estimate the 1 year (2016) period prevalence, risk factors, and clinical management for VD. The sampling frame for the current study included dogs under veterinary care within the VetCompass database for a 1‐year period from 1 January 2016 to 31 December 2016. Dogs “under veterinary care” were defined as those with either at least 1 EPR recorded from 1 January 2016 to 31 December 2016 or, alternatively, at least 1 EPR during both 2015 and 2017. Ethical approval was granted by the RVC Ethics and Welfare Committee (reference number URN 2015 1369).

Case inclusion criteria required that a final diagnosis of VD or a synonym (eg, *vestibular attack, geriatric vestibular syndrome, canine idiopathic vestibular syndrome, old‐dog vestibular disease*) was recorded in the EPR for a condition that was present during the 2016 study period. Case‐finding involved initial screening of all EPRs for candidate VD cases by searching the clinical free‐text field and VeNom term field using the search terms nyst* and vest*.

The candidate cases were randomly ordered, and the clinical notes of all candidate animals were manually reviewed in detail to evaluate for case inclusion. All dogs from the overall study denominator that were not confirmed as VD cases were included as noncases in the risk factor analysis. The full clinical notes for each confirmed vestibular case dog were manually reviewed to extract data on additional study questions of interest related to the VD: date of first diagnosis, clinical signs on first presentation, clinical management and treatments prescribed, referral, clinical improvement, and death. Options for clinical signs at presentation included the presence, absence or unrecorded status for ataxia, nystagmus, head tilt, collapse, vomiting, otitis externa, and central involvement (defined as 1 or more of the following: proprioceptive deficits, altered mentation, CN deficits other than VII or VIII, and a vertical, positional or dysconjugate nystagmus). Improvement was defined as evidence of clinical improvement and absence of ataxia and nystagmus recorded in the EPR although these dogs might still have shown a residual head tilt.

A *purebred* variable categorized all dogs of recognizable breeds as “purebred” and the remaining dogs as “crossbred.”^12^ A *breed* variable included individual breeds represented by 5 or more VD cases, a grouped category of all remaining purebreds and a general grouping of crossbred dogs. This approach was taken to facilitate statistical power for the individual breed analyses.^13^ Spaniel‐type breeds included the following breeds that had the word “spaniel” in their name: American Cocker, Brittany, Cavalier King Charles, Clumber, English Cocker, English Springer, English Toy, Field, Japanese (also known as Japanese Chin), King Charles, Picardy, Sussex, Tibetan, Welsh Cocker, Working Cocker, Water Spaniel, and Welsh Springer. Brachycephalic‐type breeds included breeds known to have brachycephalic characteristics.^14^

*Sex* (female, male, unrecorded) and *neuter* (neutered, entire, unrecorded) variables described the status recorded at the final EPR. Age (years) was calculated for all dogs at the final date of the study period (31 December 2016). An *age* variable categorized age (years) into 6 groups (<3.0, 3.0 to <6.0, 6.0 to <9.0, 9.0 to<12.0, ≥12.0 and unknown). Adult bodyweight described the maximum bodyweight (kg) recorded for dogs >18 months. An *adult bodyweight* variable categorized adult bodyweight into 5 groups (<10.0, 10.0 to <25, 25 to <40, ≥40.0 and unknown).

With the aim of reducing errors such as spelling mistakes or missing/inappropriately collected data, checking and cleaning of the data was performed in Excel (Microsoft Office Excel 2016, Microsoft Corp.). Statistical analyses were conducted using IBIM SPSS Version 26. The 1‐year period prevalence with 95% confidence intervals (CI) described the probability of having a new or ongoing diagnosis of VD at any time during the 1‐year 2016 study period. The 1‐year incidence risk with 95% CI described the probability of having a new diagnosis of VD at any time during the 1‐year 2016 study period. Descriptive statistics characterized the risk factors separately for the case and noncase dogs.

Binary logistic regression modeling was used to evaluate univariable associations between risk factors (*breed, purebred, spaniel type, brachycephalic type, adult bodyweight, age, sex, neuter*) and having a diagnosis of VD during 2016. Because breed was a factor of primary interest for the study, *purebred, spaniel type, brachycephalic type, and adult bodyweight* (a defining characteristic of individual breeds) were excluded from the initial breed multivariable modeling. Instead, each of these variables individually replaced the *breed* variable in the main final breed model in order to evaluate their effects after taking account of the other variables. In line with common practice in multivariable model building, risk factors with liberal associations in univariable modeling (*P* < .2) were taken forward for multivariable evaluation.^15^ Model development used manual backwards stepwise elimination. Pair‐wise interaction effects were evaluated for the final model variables. For the final model, the area under the receiver operating characteristic (ROC) curve was used to evaluate the quality of the model fit.^15^, ^16^ Statistical significance was set at *P* < .05.

## 3 RESULTS

The overall study population comprised 905 544 dogs under primary veterinary care at 886 veterinary clinics during 2016. Of 2600 dogs identified as candidate VD cases, manual checking identified 759 confirmed VD cases, giving an overall prevalence of 8 per 10 000 dogs (95% CI = 7‐9 per 10 000 dogs). The prevalence in dogs that were 9 years or older (636/177 754) was 36 per 10 000 dogs (95% CI = 33‐39) and the prevalence for each breed is shown in Table 1. Out of the 759 VD cases, only 39 (5.13%) were pre‐existing cases that were diagnosed with VD before 2016, therefore the incidence of VD was similar to the prevalence: 8 per 10 000 dogs (95% CI = 7‐9).

**TABLE 1:** Prevalence of vestibular disease in dogs in the VetCompass database under primary veterinary care in the United Kingdom from 1 January 2016 to 31 December 2016

	Cases	Denominator (all dogs under primary veterinary care)	Prevalence (nr per 10 000 dogs)	95% Confidence interval
Overall	759	905 544	8	7‐9
Age (y)				
0 to <3	13	329 266	0	
3 to <9	101	385 353	3	2‐4
9 to <12	110	108 475	10	8‐12
>12	526	692 279	8	7‐9
Unavailable	9	12 411	7	2‐12
Breed				
King Charles Spaniel	9	2804	32	11‐53
Springer Spaniel	63	20 145	31	23‐39
Golden Retriever	27	9766	28	18‐38
Cavalier King Charles Spaniel	39	17 219	23	16‐30
Border Collie	52	22 352	23	17‐29
Boxer	15	9427	16	8‐24
Bulldog	5	3228	15	2‐28
Miniature Schnauzer	11	8385	13	5‐21
Labrador Retriever	70	59 893	12	9‐15
Beagle	8	8062	10	3‐17
West Highland White Terrier	17	18 861	9	5‐13
German Shepherd Dog	19	21 321	9	5‐13
Cocker Spaniel	28	32 117	9	6‐12
Border Terrier	9	9642	9	3‐15
Crossbreed	168	199 798	8	7‐9
French Bulldog	11	16 386	7	3‐11
Staffordshire Bull Terrier	38	53 017	7	5‐9
Jack Russell Terrier	33	48 402	7	5‐9
Yorkshire Terrier	12	28 168	4	2‐6
Shih‐Tzu	12	32 898	4	2‐6
Other purebreds	113	282 894	4	3‐5

Of the VD cases with demographic data available, 586 (77.21%) were purebred, 381 (50.19%) were female, and 538 (70.88%) were neutered. Dogs with VD had a median adult bodyweight of 18.61 kg (interquartile range [IQR], 11.28‐26.20; range, 1.71‐79.67) and a median age at diagnosis of 12.68 years (IQR, 11.28‐14.64; range, 0.43‐20.7). The most common breeds among the VD cases and noncases are shown in Table 2.

**TABLE 2:** Descriptive and univariable logistic regression results in dogs with vestibular disease in the VetCompass database under primary veterinary care in the United Kingdom from 1 January 2016 to 31 December 2016

Variable	Category	Case no. (%)	Noncase no. (%)	Odds ratio	95% CI	*P* value
Purebred status	Crossbred	170 (22.40)	245 805 (27.14)	Base		
	Purebred	586 (77.21)	654 813 (72.31)	1.29	1.09‐1.53	.003
Spaniel type breeds	Nonspaniel type	446 (58.76)	530 052 (58.53)	Base		
	Spaniel‐type	148 (19.50)	76 784 (8.48)	1.16	0.78‐1.73	.46
	Unknown	165 (21.74)	197 949 (21.86)	1.16	0.78‐1.73	.46
Brachycephalic type breeds	Nonbrachycephalic	483 (63.64)	526 605 (58.15)	Base		
	Brachycephalic	110 (14.49)	166 793 (18.42)	0.63	0.44‐0.92	.02
	Unknown	165 (21.74%)	197 949 (21.86%)	1.11	0.49‐2.49	.79
Common breeds	Crossbreed	168 (22.13)	193 798 (21.40)	Base		
	King Charles Spaniel	9 (1.19)	2804 (0.31)	3.81	1.95‐7.47	<.001
	Springer Spaniel	63 (8.30)	20 145 (2.22)	3.71	2.78‐4.97	<.001
	Golden Retriever	27 (3.56)	9766 (1.08)	3.28	2.18‐4.93	<.001
	Border Collie	52 (6.85)	22 352 (2.47)	2.76	2.02‐3.77	<.001
	Cavalier King Charles Spaniel	39 (5.14)	17 219 (1.9)	2.69	1.90‐3.81	<.001
	Boxer	15 (1.98)	9427 (1.04)	1.89	1.11‐3.21	.02
	Bulldog	5 (0.66)	3228 (0.36)	1.84	0.75‐4.48	.18
	Miniature Schnauzer	11 (1.45)	8385 (0.93)	1.56	0.84‐2.87	.15
	Labrador Retriever	70 (9.22)	59 893 (6.61)	1.39	1.05‐1.83	.02
	Beagle	8 (1.05)	8062 (0.89)	1.18	0.58‐2.39	.65
	Border Terrier	9 (1.19)	9642 (1.06)	1.11	0.56‐2.17	.76
	West Highland White Terrier	17 (2.24)	18 861 (2.08)	1.07	0.65‐1.76	.79
	German Shepherd Dog	19 (2.50)	21 321 (2.35)	1.06	0.65‐1.70	.81
	Cocker Spaniel	28 (3.69)	32 117 (3.55)	1.03	0.69‐1.54	.86
	Staffordshire Bull Terrier	38 (5.01)	53 017 (5.85)	0.85	0.59‐1.21	.37
	Jack Russell Terrier	33 (4.35)	48 402 (5.35)	0.81	0.55‐1.17	.27
	French Bulldog	11 (1.45)	16 386 (1.81)	0.78	0.43‐1.47	.47
	Yorkshire Terrier	12 (1.58)	28 168 (3.11)	0.50	0.28‐0.91	.02
	Other purebreds	113 (14.89(	282 894 (31.24)	0.47	0.37‐0.60	<.001
	Shih‐Tzu	12 (1.58)	32 898 (3.63)	0.43	0.24‐0.77	.005
Adult (over 18 mo.) bodyweight (kg)	0 to <10	98 (12.91)	213 352 (23.56)	Base		
	10 to <25	303 (39.92)	231 667 (25.58)	1.72	1.33‐2.22	<.001
	25 to <40	131 (17.26)	123 724 (13.66)	1.33	0.98‐1.81	.64
	≥40	19 (2.50)	26 257 (2.90)	1.39	0.82‐2.35	.21
	Unknown	208 (27.40)	310 542 (34.29)	1.66	1.28‐2.16	<.001
Age category (y)	0 to <3	13 (1.71)	329 266 (36.36)	Base		
	3 to <6	36 (4.74)	222 304 (24.54)	4.14	2.01‐8.53	<.001
	6 to <9	65 (8.56)	163 049 (18.00)	12.67	6.49‐24.71	<.001
	9 to <12	110 (14.49)	108 475 (11.97)	32.93	17.22‐62.94	<.001
	≥12	526 (69.30)	692 279 (76.45)	255.92	136.90‐478.40	<.001
	Unknown	9 (1.18)	12 411 (1.37)	17.46	7.35‐41.46	<.001
Sex	Female	381 (50.20)	431 328 (47.63)	Base		
	Male	372 (49.01)	469 233 (51.82)	0.97	0.84‐1.12	.69
	Unknown	6 (0.79)	4222 (0.47)	0	0‐0	1.000
Neuter status	Entire	215 (38.33)	493 196 (54.46)	Base		
	Neutered	538 (70.88)	407 427 (44.99)	1.54	1.31‐1.81	.33
	Unknown	6 (0.79)	4222 (0.47)	0	0‐0	1.000

Of the noncase dogs with complete data available, 654 813 (72.31%) were purebred, 431 328 (47.63%) were female, and 407 427 (44.99%) were neutered. The median adult bodyweight for noncases was 16.94 kg (IQR, 9.08‐22.10; range, 1.37‐79). Data completeness across the variables assessed was breed 99.91%, age 98.62%, sex 99.53%, neuter status 99.53%, and adult bodyweight 65.68%.

Univariable logistic regression modeling identified 4 variables that were liberally associated with VD and were further evaluated in the breed multivariable logistic regression modeling: breed, adult bodyweight, purebred status, and age. Sex and neuter status were not associated with the odds of VD in univariable analysis.

The final breed multivariable model retained 2 variables: breed and age. Compared with crossbred dogs, 11 breeds showed increased odds of VD after accounting for the confounding effect of age. The final model showed acceptable discrimination (area under the ROC curve: 0.662). The breeds with the highest odds included the French Bulldog (odds ratio [OR] = 9.25, 95% CI = 4.81‐17.76, *P* < .001), Bulldog (OR = 6.56, 95% CI = 2.66‐16.15, *P* < .001), King Charles Spaniel (OR = 4.96, 95% CI = 2.52‐9.78, *P* < .001), Cavalier King Charles Spaniel (CKCS) (OR = 3.56, 95% CI = 2.50‐5.06, *P* < .001), Springer Spaniel (OR = 3.37, 95% CI = 2.52‐4.52, *P* < .001), Boxer (OR = 2.68, 95% CI = 1.57‐4.56, *P* < .001), and Golden Retriever (OR = 2.55, 95% CI = 1.69‐3.84, *P* < .001) (Table 3).

**TABLE 3:** Final multivariable logistic regression results in dogs with VD in the VetCompass database under primary veterinary care in the United Kingdom from 1 January 2016 to 31 December 2016

Variable	Category	Odds ratio	95% CI	*P* value	Median age (y) (range)
Common breeds	Crossbreed	Base			13.67 (2.75‐17.83)
	French Bulldog	9.25	4.81‐17.76	<.001	5.66 (1.06‐18.14)
	Bulldog	6.56	2.66‐16.15	<.001	4.42 (1.99‐9.46)
	King Charles Spaniel	4.96	2.52‐9.78	<.001	10.76 (5.10‐14.67)
	Cavalier King Charles Spaniel	3.56	2.50‐5.06	<.001	8.83 (2.61‐14.36)
	Springer Spaniel	3.37	2.52‐4.52	<.001	13.67 (4.46‐15.78)
	Boxer	2.68	1.57‐4.56	<.001	9.75 (6.07‐16.55)
	Golden Retriever	2.55	1.69‐3.84	<.001	14.10 (3.85‐15.41)
	Beagle	2.52	1.23‐5.14	.01	10.40 (6.84‐14.25)
	Miniature Schnauzer	2.34	1.26‐4.33	.007	13.62 (3.01‐15.60)
	Border Collie	2.16	1.58‐2.96	<.001	14.06 (11.43‐16.58)
	German Shepherd Dog	1.52	0.94‐2.45	.08	12.43 (5.83‐16.91)
	Labrador Retriever	1.36	1.03‐1.81	.03	13.63 (1.00‐17.16)
	Cocker Spaniel	1.32	0.88‐1.97	.17	13.58 (2.59‐17.67)
	Border Terrier	0.99	0.50‐1.94	.98	13.81 (10.97‐16.03)
	Other purebreds	0.81	0.63‐1.03	.09	12.60 (0.43‐18.45)
	Staffordshire Bull Terrier	0.78	0.55‐1.12	.19	14.12 (5.45‐20.07)
	Shih‐Tzu	0.71	0.39‐1.28	.26	12.69 (1.73‐17.07)
	West Highland White Terrier	0.58	0.35‐0.96	.04	13.91 (1.02‐17.99)
	Jack Russell Terrier	0.56	0.38‐0.81	.003	14.60 (3.36‐29.24)
	Yorkshire Terrier	0.39	0.21‐0.70	.002	12.62 (5.97‐17.21)
Age category (y)	0 to <3	Base			
	≥12	327.71	171.73‐625.38	<.001	
	9 to <12	38.82	19.93‐75.59	<.001	
	Unknown	24.76	10.05‐60.93	<.001	
	6 to <9	14.88	7.51‐29.49	<.001	
	3 to <6	7.63	2.28‐9.80	<.001	

Abbreviation: VD, vestibular disease.

The odds of VD increased significantly with age; dogs over 12 years of age had 327.71 times the odds (95% CI = 171.73‐625.38) and dogs between 9 and 12 years of age had 38.82 times the odds (95% CI = 19.93‐75.59 of VD compared to dogs under 3 years of age). The breeds that were less likely to be diagnosed with VD were West Highland White Terrier (OR = 0.58, 95% CI = 0.35‐0.96, *P* = .04), Jack Russell Terrier (OR = 0.56, 95% CI = 0.38‐0.81, *P* = .003), and Yorkshire Terrier (OR = 0.39, 95% CI = 0.21‐0.70, *P* = .002).

Spaniel type breeds were strongly associated with VD, showing 1.98 times the odds (95% CI = 1.61‐2.43, *P* < .001) compared with nonspaniel type breeds. Brachycephalic type breeds had 1.29 times the odds of VD compared with nonbrachycephalic type breeds (95% CI = 1.02‐1.63, *P* = .03).

Dogs with an adult bodyweight under 10 kg were significantly less likely to be diagnosed with VD when compared to all other bodyweight categories.

The most common presenting signs recorded for VD cases were head tilt (530/759, 69.82%), nystagmus (516/759, 67.98%), and ataxia (489/759, 64.42%) (Table 4). The direction of nystagmus was recorded in 371/516 (71.89%) cases, with most cases reported to have a horizontal nystagmus (304/516, 58.91%), out of which 70/304 (23.02%) cases had a horizontal nystagmus with the fast phase to the left, 67/304 (22.03%) had a fast phase to the right, and 167/304 (54.93%) were recorded as horizontal only. A vertical nystagmus was noted in 38/516 (7.36%) cases, a rotary nystagmus in 28/516 (5.42%) cases and 1 case had a description consistent with dysconjugate nystagmus (each eye moving in a different direction).

**TABLE 4:** Multivariable logistic regression results for variables that replaced breed in dogs with VD in the VetCompass database under primary veterinary care in the United Kingdom from 1 January 2016 to 31 December 2016

Variable	Category	Odds ratio	95% CI	*P* value
Purebred status	Crossbred	Base		
	Purebred	0.98	0.55‐1.76	.97
Spaniel type breeds	Nonspaniel type	Base		
	Spaniel‐type	1.98	1.61‐2.43	<.001
Brachycephalic type breeds	Nonbrachycephalic	Base		
	Brachycephalic	1.29	1.02‐1.63	.03
Adult (over 18 mo.) bodyweight (kg)	0‐10	Base		
	10 to <25	2.26	1.78‐2.87	<.001
	25 to <40	2.21	1.69‐2.89	<.001
	≥40	2.13	1.30‐3.50	.003
	Unavailable	2.12	1.66‐2.72	<.001
Age category (y)	0 to <3	Base		
	≥12	267.35	142.35‐502.10	<.001
	9 to <12	32.71	17.03‐62.83	<.001
	Unavailable	24.03	9.73‐59.30	<.001
	6 to <9	12.79	6.53‐25.06	<.001
	3 to <6	4.35	2.10‐8.99	<.001

Abbreviation: VD, vestibular disease.

Of 530 dogs that presented with a head tilt, 232 (43.77%) had a left sided head tilt, 208 (39.24%) had a right sided head tilt, and in 90 (16.98%) cases the head tilt direction was not recorded. From the cases where both the direction of the horizontal nystagmus and the head tilt were recorded, 66/168 (39.28%) had a left sided head tilt with a horizontal nystagmus with a fast phase to the right side, and 102/168 (60.71%) had a right sided head tilt with a horizontal nystagmus with a fast phase to the left side.

Out of the 759 cases diagnosed with VD, 139 (18.31%) were hospitalized for management and the treatments prescribed are summarized in Table 5.

**TABLE 5:** Description of vestibular disease cases in 759 dogs in the VetCompass database under primary veterinary care in the United Kingdom from 1 January 2016 to 31 December 2016

	Categories	N (%)
Clinical signs	Head tilt	530 (69.82)
	Nystagmus	516 (67.98)
	Ataxia	489 (64.42)
	Collapse	244 (32.14)
	Vomiting	195 (25.69)
	Otitis externa	119 (15.67)
	Central involvement	107 (14.09)
Treatments prescribed	Antiemetics	328 (43.21)
	Steroids	251 (33.06)
	Antibiotics	190 (25.03)
	Propentofylline	176 (23.18)
	Intravenous fluid treatment	98 (12.91)
	Nonsteroidal anti‐inflammatories	75 (9.88)
	Sedation	24 (3.16)
Antiemetic type	Maropitant	302 (39.78)
	Prochlorperazine	13 (1.71)
	Metoclopramide	9 (1.18)
	Maropitant + prochlorperazine	1 (0.13)
	Maropitant + metoclopramide	1 (0.13)
Antimicrobial type	Amoxicillin and clavulanic acid	108 (14.22)
	Enrofloxacin	36 (4.74)
	Marbofloxacin	11 (1.44)
	Cefalexin	10 (1.31)
	Cefovecin	6 (0.79)
	Clindamycin	6 (0.79)
	Pradofloxacin	6 (0.79)
	Cefuroxime	4 (0.52)
	Doxycycline	1 (0.13)
	Metronidazole	1 (0.13)
	Enrofloxacin + amoxiclav	1 (0.13)
	Enrofloxacin + marbofloxacin	1 (0.13)
Sedative type	Diazepam	13 (1.71)
	Medetomidine	3 (0.39)
	Midazolam	2 (0.26)
	Hydroxyzine dihydrochloride	1 (0.13)
	Diazepam + acepromazine	1 (0.13)
Outcomes	Improvement recorded	316 (41.63)
	Lost to follow‐up	310 (40.84)
	Death during the study period	232 (30.56)
	Death due to vestibular disease during the study period	144 (18.97)
	Euthanasia at the same time with diagnosis	88 (11.59)
	Recurrence	78 (10.27)
	Definite diagnosis	35 (3.29)
	Referred	27 (3.55)

There were 27/759 (3.55%) cases referred. A specific cause of VD in the clinical notes or in a referral letter was recorded in 33/759 (4.34%) of the cases. Out of these, 14 were recorded with IPVD, 11 with brain disease, 7 with either otitis media or interna, and 1 case with metronidazole toxicity. Of the 14 cases diagnosed with IPVD, 3/14 (21.42%) were reported to have facial nerve palsy.

Of the 11 cases with a brain disease confirmed, 6 were diagnosed with meningoencephalitis of unknown origin (MUO), 2 with intracranial neoplasia, 1 with Chiari‐like malformation, 1 with rostral cerebellar ischemic stroke (suspected to be caused by thromboembolic disease), and 1 with an unspecified cerebellar lesion.

Out of the 7 cases that were diagnosed with otitis media or interna, 1 case was reported to have Horner's syndrome, and another had Horner's syndrome with facial nerve palsy.

Clinical improvement was recorded in 317/759 (41.76%) of the cases after a median 4 days (IQR, 2‐10.25; range, 0‐160 days) from the first diagnosis. In 310/759 (40.84%) of the cases, there was no mention of improvement or deterioration because of the loss of complete follow‐up for VD. A persistent head tilt (defined as head tilt recorded in the EPR after more than 1 month from improvement) was recorded in 68 (8.95%) of the cases and recurrence of VD was recorded in 78 (10.27%) of the cases.

Of 232/759 (30.56%) deaths during the study period, there was evidence in the EPRs that VD contributed to the death or the decision to euthanize in 144/232 (62.06%) of the deaths and 144/759 (18.97%) of the total cases. Out of these, 88/759 (11.59%) were euthanized on first veterinary presentation with VD.

## 4 DISCUSSION

This is the largest study to explore the wider presentation of VD in dogs attending primary‐care practices by analyzing clinical data from a multicenter primary‐care research database. Previous studies of VD^5^, ^7^, ^17^, ^18^, ^19^ have relied mainly on referral populations and therefore might be poorly representative of the general population. The overall prevalence of VD in the current study was 8 per 10 000 dogs, but the prevalence in dogs aged 9 years or older was significantly higher at 36 per 10 000 dogs.

French Bulldogs and Bulldogs had the highest odds of diagnosis with VD compared to crossbreed dogs: 9.25 and 6.56 times, respectively. French bulldogs have previously been reported as more frequently affected by VD compared to other breeds.^5^ In a United Kingdom‐based referral population of French Bulldogs that presented with neurological disorders, 16/343 were diagnosed with peripheral VD and 25/343 were diagnosed with brain neoplasia.^20^ Brachycephalic breeds are reported to have a higher incidence of gliomas that can cause vestibular signs.^21^ MUO and otitis media and/or interna are also common in French Bulldogs and these conditions can present showing vestibular signs.^20^ These findings add to the already known disease burden^22^, ^23^, ^24^, ^25^, ^26^, ^27^, ^28^ for French Bulldogs and Bulldogs and have important welfare implications, as VD can cause disorientation, loss of balance, and even pain in cases of otitis media/interna. A previous study of the overall disorders in French Bulldogs under primary care in the United Kingdom reported “brain disorder” as the most common cause of mortality.^26^ It is interesting to note that both French Bulldogs and Bulldogs did not show significantly increased odds for being diagnosed with VD in the univariable logistic regression analysis (Table 2). We believe that, in the univariable model, the age was a confounding factor. Both breeds had a lower median age at presentation (Table 3), which could suggest that diseases associated with older age (such as IPVD or neoplastic diseases) were less likely to be the cause for VD in these breeds.

King Charles Spaniels and CKCS were also shown to have increased odds of VD compared with crossbred dogs, 4.96 and 3.56 times, respectively. A previous study on the disorders causing presentation to primary care practice in CKCS found a prevalence of neurological causes to be 4.3%.^29^ CKCS are known to be predisposed to several neurological syndromes that can cause vestibular signs, including occipital hypoplasia/syringomyelia, granulomatous meningoencephalomyelitis (GME), and VD.^30^ Moreover, CKCS have a tendency toward cerebrovascular disease, particularly rostral cerebellar artery infarction.^31^, ^32^, ^33^ In immature dogs, the first presenting sign of syringomyelia could be scoliosis, which can appear similar to a head tilt of vestibular origin.^30^ VD in CKCS can be also idiopathic—CKCS is one of the most commonly seen breeds with idiopathic facial nerve paralysis^34^ and facial and vestibular neuropathy of unknown origin.^35^ Aside from central and IPVD, CKCS are also predisposed to primary secretory otitis media, known to commonly be associated with peripheral VD.^3^, ^36^

Springer spaniels were shown to have 3.37 times the odds of diagnosis with VD compared to crossbreds. Springer Spaniels are predisposed to cerebellar infarcts that occur most commonly within the region supplied by the rostral cerebellar artery^33^ and to immune mediated hemolytic anemia (IMHA)^37^ that is often associated with a hypercoagulable state. VD has been reported as a complication of IMHA.^38^

As expected, considering the breed predispositions, spaniel type and brachycephalic type breeds were strongly associated with VD.

Dogs under 10 kg are substantially less likely to be affected by VD compared with dogs of all other sizes. West Highland White, Jack Russell, and Yorkshire Terries are all small breeds and are shown to have decreased odds of VD in our study. Smaller breeds are known to have increased longevity^39^ due to genetic factors that potentially contribute to the lower risk of VD. Moreover, large breed dogs have an increased oxidative damage and a faster rate of aging compared to smaller breeds^40^ and this could potentially contribute to an increased predisposition toward IPVD or other causes of VD.

In our study, the median age at first diagnosis was 12.68 years. This suggests that IPVD might be implicated in most cases, as the mean age of onset for IPVD in published literature is between 9.4 and 12.5 years.^5^, ^18^, ^21^ Other causes of VD cannot be excluded, however, as it has been documented that the incidence of brain tumors, for example, is higher in dogs older than 5 years.^41^ Regardless of the primary cause, VD should be considered a high‐risk disease in older dogs, especially in the predisposed breeds described above.

The most common clinical signs on presentation were ataxia, nystagmus, and head tilt, which are clinical signs that are already known as the hallmark signs of VD.^42^ Vertical nystagmus is generally present only in central VD^1^, ^3^ and was documented in 7.3% of the cases that presented with nystagmus. This should be interpreted with caution however, as vertical nystagmus can be difficult to differentiate from a rotary one (which can be seen in either peripheral or central VD). The presence of pathological nystagmus tends to be short‐lived, especially in cases of peripheral VD, because it can often be rapidly compensated by voluntary visual fixation.^3^ The high prevalence of nystagmus among the VD cases in our study therefore suggests an acute presentation.

Vomiting is a common clinical sign of VD in dogs^43^ and in our study 25.7% of cases presented with vomiting, similar to previously published literature.^18^ Although vomiting occurs with VD regardless of the cause, it is more common in dogs with acute peripheral VD and is a result of the connection between the vestibular nuclei axons and the vomiting center in the brainstem reticular formation.^3^, ^21^ Vestibular disorders in people are known to cause unpleasant autonomic signs such as nausea and vomiting that can lead to anxiety and panic attacks.^44^ Even though vomiting is usually transitory in dogs with VD,^21^ it should therefore be considered to have welfare implications.

Collapse was recorded in 32.1% of the cases, but it is difficult to discern whether this was due to central disease and loss of proprioception or due to marked imbalance and disorientation. We therefore suspect the presence of collapse is an indication of the severity of the disease or the acute onset.

Differentiating between central and peripheral VD is an important goal for the clinician when assessing a dog with signs of VD. Central VD generally requires more expense to diagnose and treat, and can be associated with a poorer prognosis.^45^ In our study, signs of central involvement were identified in 14.1% of cases. However, the true proportion with central VD could be higher as many cases did not have a full neurological examination described in the EPRs.

Several disorders can cause peripheral VD but the most common ones are IPVD and otitis media/interna.^2^ A study of 85 dogs with peripheral VD referred for advanced imaging reported that 74% had magnetic resonance imaging abnormalities^7^ but in our study only a small percentage (3.6%) of the cases were referred. Otitis media is diagnosed in 49% and 41% of peripheral VD cases^7^, ^18^ although these studies were both based on cases seen in specialist centers.

Otitis externa was recorded in 15.7% of the vestibular cases in our study but it is possible that undiagnosed otitis externa was present in further cases. Otitis media/interna is reported as the most common cause of peripheral VD in dogs and is a common complication of otitis externa.^3^, ^46^ A careful otoscopic examination should therefore be performed when examining a dog with VD,^47^ even though a normal tympanic membrane does not rule out the presence of otitis media/interna.^3^

The presence of facial nerve paralysis or Horner's disease usually indicates an underlying cause of peripheral vestibular dysfunction such as otitis media/interna,^48^ therefore documenting these deficits can help veterinary practitioners in differentiating between central and peripheral VD. Moreover, cases with idiopathic facial nerve paralysis can present with vestibular signs.^34^ In our study, 2/7 cases diagnosed with otitis media or interna had Horner's syndrome reported (1 of which also had facial nerve palsy) and 3/14 cases diagnosed with IPVD had facial nerve palsy reported.

Only 27 (3.6%) of the cases were referred. We suspect this is related to the high rate of improvement noted in these cases, but owner preference, financial constraints, age of the dog, guarded prognosis, and veterinary recommendations might play a role. Either way, this low proportional referral confirms that, for VD in dogs, studies performed on referral caseloads will have limited generalizability to the wider primary‐care population because of selection bias and suggest that epidemiological studies based on large primary‐care populations are needed to accurately benchmark primary‐care clinical activity.^49^

A formal cause of VD was recorded in only 32 (4.2%) cases. Most of the cases that had either brain disease, IPVD, otitis media, or interna confirmed were diagnosed in a referral center (19/32, 59%), although some of the primary‐care practices were capable of performing advanced diagnostic imaging in‐house in 13/32 (49%) of the cases.

Because of the low proportion of referral and hence the limited access to advanced imaging for diagnostic investigation, this limited our ability to differentiate between the different causes of VD. Consequently, the percentage of cases with vestibular signs caused by IPVD, for example, remains unknown. Since only a small percentage had signs of central involvement on presentation, it is likely that most cases were characterized by peripheral vestibular dysfunction. This is contradictory to the findings based on referral populations of VD cases, where 62% to 86% of the VD cases were diagnosed with central VD,^5^, ^19^ possibly because general practitioners might refer more severe and central VD cases.

In our study, taking into consideration the signalment and presentation of the vestibular cases as well as the significant rate of improvement, we suspected most cases were caused by IPVD. Clinical signs associated with IPVD usually improve in 1 to 2 weeks and no treatment has been proved beneficial.^4^, ^50^

The most commonly used treatments for VD in our study were antiemetics, systemic glucocorticoids, and antibiotics. Vomiting in dogs with VD is similar to motion sickness and is present especially in cases of acute vestibular dysfunction.^1^ Maropitant was the most commonly used antiemetic and it has been showed to be effective in the prevention of vomiting induced by motion sickness in dogs.^51^

Systemic treatment with antimicrobials based on culture and sensitivity is the medical treatment of choice for otitis media/interna.^2^ However, in our study, only 7 cases had otitis media/interna confirmed. Moreover, only 15.7% of the cases had otitis externa documented and in 46.1% of the cases there was no mention of otoscopic examination. Therefore, we suspected that many of the 25% cases that were prescribed antibiotics were receiving these as an empirical treatment. From the dogs treated with antibiotics, most received amoxicillin potentiated with clavulanic acid (56.8%) although there is an important proportion of cases (27.9%) that received fluoroquinolones. Compared to the most commonly used antimicrobials in first opinion practice in the United Kingdom, where fluoroquinolones were prescribed in 4.84% of the cases that received antimicrobials,^52^ fluoroquinolones are used more commonly in VD. The total use of antimicrobials classified as critically important (CIAs) with highest priority for human medicine^53^ in VD cases was 28.7% of the cases that received antimicrobial treatment, compared to 6.4% in dogs that received antimicrobial treatment for all conditions in primary care practices in United Kingdom in from 2012 to 2014.^52^

Similarly, although the use of systemic glucocorticoids is recommended in cases of inflammatory brain disease and possibly in cases of otitis media/interna, it is likely that a large proportion of the 33.1% cases treated with glucocorticoids were treated empirically. In cases of IPVD, there is no evidence that anti‐inflammatory treatment with glucocorticoids is beneficial.^3^ In addition, considering that glucocorticoids commonly cause adverse effects especially in older dogs,^54^, ^55^ glucocorticoid use should be limited to the cases with an underlying cause where these treatments might be beneficial.

Improvement was recorded in 41.8% of the cases after a median 4 days but this ranged from 0 to 160 days from the first diagnosis. In most cases that had a prolonged time to improvement documented, this was likely because of delayed follow‐up rather than slow improvement. We therefore believe that the time to improvement might be substantially shorter than reported here. Considering the strict definition used for improvement and that in 40.7% of the cases there was no mention of improvement or deterioration due to loss of follow‐up, we suspect the great majority of cases improved. A lack of improvement was noted in only 16.4% of the cases.

A large number of cases (11.5%) were euthanized on presentation and where VD contributed to the decision. This could be due to veterinary recommendations or at the request of the owner. VD cases can show a dramatic presentation causing a lot of distress to the owners. Also, considering that the median age at diagnosis was quite old, other concurrent diseases could be contributing to the decision of euthanasia. However, it is possible that owners could now be reassured by their veterinary surgeons about a good probability of improvement reported in both the previous literature as well as the current study and might opt to trial treatment which is generally affordable and to monitor for signs of recovery for at least a few days after diagnosis in a greater proportion of cases.

The limitations of the study mainly arise from the retrospective design. The data collected from the EPRs were not recorded primarily for research purposes and were therefore limited by incomplete information and potential lack of accuracy. We suspect the prevalence of 8 per 10 000 dogs in our study is underreporting the prevalence of VD in primary‐care clinical practice. By using the search terms “nyst” and “vest,” we retrieved 2600 potential cases and we manually evaluated each against the case definition, but potential true cases could have been missed due to misspelling or lack of complete entry of clinical signs in the EPRs by the veterinary surgeon. Moreover, the case definition for inclusion in the study relied on the veterinary surgeon stating *vestibular disease* or a synonym but did not include other terms commonly used by veterinary surgeons such as *stroke*, *vascular accident*, or similar. Using search terms like *nyst* to identify potential cases likely introduced a bias toward the prevalence of nystagmus among VD cases. The study included all cases of VD and did not attempt to categorize these based on central/peripheral dysfunction or based on etiology. A neurological examination was not recorded in most of the cases, making the distinction between peripheral and central VD impossible.

## CONFLICT OF INTEREST DECLARATION

Authors declare no conflict of interest.

## OFF‐LABEL ANTIMICROBIAL DECLARATION

Authors declare no off‐label use of antimicrobials.

## INSTITUTIONAL ANIMAL CARE AND USE COMMITTEE (IACUC) OR OTHER APPROVAL DECLARATION

Royal Veterinary College Ethics and Welfare Committee (reference number URN 2015 1369).

## HUMAN ETHICS APPROVAL DECLARATION

Authors declare human ethics approval was not needed for this study.

## References

[1] Sanders SG . Disorders of hearing and balance: the vestibulochochlear nerve (CN VIII) and associated structures In: Dewey CW, Costa RC, eds. A Practical Guide to Canine and Feline Neurology. 3rd ed. Ames, IA: Wiley‐Blackwell; 2016:277‐297.

[2] Kent M , Platt SR , Schatzberg SJ . The neurology of balance: function and dysfunction of the vestibular system in dogs and cats. Vet J. 2010;185(3):247‐258. 19944632 10.1016/j.tvjl.2009.10.029

[3] Rossmeisl JH . Vestibular disease in dogs and cats. Vet Clin North Am Small Anim Pract. 2010;40(1):81‐100. 19942058 10.1016/j.cvsm.2009.09.007

[4] Garosi LS . Head tilt and nystagmus In: Platt SR, Garosi LS, eds. Small Animal Neurological Emergencies. London: Manson Publishing; 2012:253‐263.

[5] Bongartz U , Nessler J , Maiolini A , Stein VM , Tipold A , Bathen‐Nöthen A . Vestibular disease in dogs: association between neurological examination, MRI lesion localisation and outcome. J Small Anim Pract. 2020;61(1):57‐63. 31515806 10.1111/jsap.13070

[6] Fluehmann G , Doherr MG , Jaggy A . Canine neurological diseases in a referral hospital population between 1989 and 2000 in Switzerland. J Small Anim Pract. 2006;47(10):582‐587. 17004950 10.1111/j.1748-5827.2006.00106.x

[7] Garosi LS , Dennis R , Penderis J , et al. Results of magnetic resonance imaging in dogs with vestibular disorders: 85 cases (1996‐1999). J Am Vet Med Assoc. 2001;218(3):385‐391. 11201565 10.2460/javma.2001.218.385

[8] Bartlett PC , Van Buren JW , Neterer M , Zhou C . Disease surveillance and referral bias in the veterinary medical database. Prev Vet Med. 2010;94(3–4):264‐271. 20129684 10.1016/j.prevetmed.2010.01.007

[9] O′Neill DG , Church DB , McGreevy PD , et al. Prevalence of disorders recorded in dogs dttending primary‐care veterinary practices in England. PLoS One. 2014;9(3):e90501. 24594665 10.1371/journal.pone.0090501 PMC3942437

[10] VeNom . The VeNom Coding Group [Internet]. VeNom Veterinary Nomenclature [Online]. http://www.venomcoding.org/VeNom/VeNom_Codes.html. Accessed November 27, 2018.

[11] O'Neill DG , Scudder C , Faire JM , et al. Epidemiology of hyperadrenocorticism among 210,824 dogs attending primary‐care veterinary practices in the UK from 2009 to 2014. J Small Anim Pract. 2016;57(7):365‐373. 27279104 10.1111/jsap.12523

[12] Irion DN , Schaffer AL , Famula TR , Eggleston ML , Hughes SS , Pedersen NC . Analysis of genetic variation in 28 dog breed populations with 100 microsatellite markers. J Hered. 2003;94(1):81‐87. 12692167 10.1093/jhered/esg004

[13] Scott M , Flaherty D , Currall J . Statistics: how many? J Small Anim Pract. 2012;53(7):372‐376. 22747729 10.1111/j.1748-5827.2012.01231.x

[14] Breed Information Centre from the Kennel Club [Internet]. https://www.thekennelclub.org.uk/services/public/breed/Default.aspx. Accessed November 27, 2018.

[15] Dohoo IR , Martin SW , Stryhn H . Veterinary Epidemiologic Research. Charlotte, Canada: VER, Inc; 2009:865.

[16] Hosmer DW , Lemeshow S . Applied Logistic Regression. 2nd ed. Hoboken, NJ: John Wiley & Sons; 2004:392.

[17] Troxel MT , Drobatz KJ , Vite CH . Signs of neurologic dysfunction in dogs with central versus peripheral vestibular disease. J Am Vet Med Assoc. 2005;227(4):570‐574. 16117064 10.2460/javma.2005.227.570

[18] Schunk KL , Averill DR . Peripheral vestibular syndrome in the dog: a review of 83 cases. J Am Vet Med Assoc. 1983;182(12):1354‐1357. 6603454

[19] Boudreau CE , Dominguez CE , Levine JM , et al. Reliability of interpretation of neurologic examination findings for the localization of vestibular dysfunction in dogs. J Am Vet Med Assoc. 2018;252(7):830‐838. 29553894 10.2460/javma.252.7.830

[20] Mayousse V , Desquilbet L , Jeandel A , Blot S . Prevalence of neurological disorders in French bulldog: a retrospective study of 343 cases (2002–2016). BMC Vet Res. 2017;13(1):212. 28676057 10.1186/s12917-017-1132-2 PMC5497356

[21] Schunk KL . Disorders of the vestibular system. Vet Clin North Am Small Anim Pract. 1988;18(3):641‐665. 3289249 10.1016/s0195-5616(88)50060-8

[22] O'Neill DG , Jackson C , Guy JH , et al. Epidemiological associations between brachycephaly and upper respiratory tract disorders in dogs attending veterinary practices in England. Canine Genet Epidemiol. 2015;2(1):10. 26401338 10.1186/s40575-015-0023-8 PMC4579368

[23] O'Neill DG , Lee MM , Brodbelt DC , Church DB , Sanchez RF . Corneal ulcerative disease in dogs under primary veterinary care in England: epidemiology and clinical management. Canine Genet Epidemiol. 2017;4(1):5. 28630713 10.1186/s40575-017-0045-5 PMC5471714

[24] O'Neill DG , Meeson RL , Sheridan A , Church DB , Brodbelt DC . The epidemiology of patellar luxation in dogs attending primary‐care veterinary practices in England. Canine Genet Epidemiol. 2016;3(1):4. 27280025 10.1186/s40575-016-0034-0 PMC4898461

[25] O'Neill DG , O'Sullivan AM , Manson EA , et al. Canine dystocia in 50 UK first‐opinion emergency care veterinary practices: clinical management and outcomes. Vet Rec. 2019;184(13):409. 30718270 10.1136/vr.104944

[26] O'Neill DG , Baral L , Church DB , Brodbelt DC , Packer RMA . Demography and disorders of the French Bulldog population under primary veterinary care in the UK in 2013. Canine Genet Epidemiol. 2018;5(1):3. 29750111 10.1186/s40575-018-0057-9 PMC5932866

[27] Packer RMA , Hendricks A , Tivers MS , Burn CC . Impact of facial conformation on canine health: brachycephalic obstructive airway syndrome. PLoS One. 2015;10(10):e0137496. 26509577 10.1371/journal.pone.0137496 PMC4624979

[28] O'Neill DG , Skipper AM , Kadhim J , Church DB , Brodbelt DC , Packer RMA . Disorders of Bulldogs under primary veterinary care in the UK in 2013. PLoS One. 2019;14(6):e0217928. 31188857 10.1371/journal.pone.0217928 PMC6561557

[29] Summers JF , O'Neill DG , Church DB , Thomson PC , McGreevy PD , Brodbelt DC . Prevalence of disorders recorded in Cavalier King Charles Spaniels attending primary‐care veterinary practices in England. Canine Genet Epidemiol. 2015;2(1):4. 26401332 10.1186/s40575-015-0016-7 PMC4579365

[30] Rusbridge C . Neurological diseases of the Cavalier King Charles spaniel. J Small Anim Pract. 2005;46(6):265‐272. 15971896 10.1111/j.1748-5827.2005.tb00319.x

[31] Garosi L , McConnell JF , Platt SR , et al. Results of diagnostic investigations and long‐term outcome of 33 dogs with brain infarction (2000‐2004). J Vet Intern Med. 2005;19(5):725‐731. 16231718 10.1892/0891-6640(2005)19[725:rodial]2.0.co;2

[32] Thomsen B , Garosi L , Skerritt G , et al. Neurological signs in 23 dogs with suspected rostral cerebellar ischaemic stroke. Acta Vet Scand. 2016;58(1):40. 27267355 10.1186/s13028-016-0219-2 PMC4897939

[33] McConnell JF , Garosi L , Platt SR . Magnetic resonance imaging findings of presumed cerebellar cerebrovascular accident in twelve dogs. Vet Radiol Ultrasound. 2005;46(1):1‐10. 15693551 10.1111/j.1740-8261.2005.00001.x

[34] Chan MK , Toribio JALML , Podadera JM , Child G . Incidence, cause, outcome and possible risk factors associated with facial nerve paralysis in dogs in a Sydney population (2001–2016): a retrospective study. Aust Vet J. 2020;98(4):140‐147. 31867719 10.1111/avj.12906

[35] Jeandel A , Thibaud JL , Blot S . Facial and vestibular neuropathy of unknown origin in 16 dogs. J Small Anim Pract. 2016;57(2):74‐78. 27168487 10.1111/jsap.12428

[36] Cole LK . Primary secretory otitis media in Cavalier King Charles spaniels. Vet Clin North Am Small Anim Pract. 2012;42(6):1137‐1142. 23122173 10.1016/j.cvsm.2012.08.002

[37] McAlees T . Immune‐mediated haemolytic anaemia in 110 dogs in Victoria, Australia. Aust Vet J. 2010;88(1‐2):25‐28. 20148823 10.1111/j.1751-0813.2009.00537.x

[38] West LD , Hart JR . Treatment of idiopathic immune‐mediated hemolytic anemia with mycophenolate mofetil in five dogs. J Vet Emerg Crit Care. 2014;24(2):226‐231. 10.1111/vec.12121 24251650

[39] O'Neill DG , Church DB , McGreevy PD , et al. Longevity and mortality of owned dogs in England. Vet J. 2013;198(3):638‐643. 24206631 10.1016/j.tvjl.2013.09.020

[40] Salvin HE , McGreevy PD , Sachdev PS , Valenzuela MJ . The effect of breed on age‐related changes in behavior and disease prevalence in cognitively normal older community dogs, *Canis lupus familiaris* . J Vet Behav. 2012;7(2):61‐69.

[41] Bagley RS , Gavin PR , Moore MP , Silver GM , Harrington ML , Connors RL . Clinical signs associated with brain tumors in dogs: 97 cases (1992‐1997). J Am Vet Med Assoc. 1999;215(6):818‐819. 10496135

[42] Lorenz MD , Coates JR , Kent M . Handbook of Veterinary Neurology. St. Louis, MO: Elsevier/Saunders; 2011:545.

[43] Thomas WB . Vestibular dysfunction. Vet Clin North Am Small Anim Pract. 2000;30(1):227‐249. 10680217 10.1016/s0195-5616(00)50011-4

[44] Brandt T . Management of vestibular disorders. J Neurol. 2000;247(7):491‐499. 10993488 10.1007/s004150070146

[45] Lowrie M , Vetmb M . Vestibular disease: anatomy, physiology, and clinical signs. Compend Contin Educ Vet. 2012;34(7):E1. 22847320

[46] Lowrie M , Vetmb MA . Vestibular disease: diseases causing vestibular signs. Compend Contin Educ Vet. 2012;34(7):E2. 22847321

[47] Platt SR , Jaggy A . Small Animal Neurology: An Illustrated Text. Hannover: Schlütersche; 2010:580.

[48] Garosi LS , Lowrie ML , Swinbourne NF . Neurological manifestations of ear disease in dogs and cats. Vet Clin North Am Small Anim Pract. 2012;42(6):1143‐1160. 23122174 10.1016/j.cvsm.2012.08.006

[49] O'Neill DG , Church DB , McGreevy PD , et al. Approaches to canine health surveillance. Canine Genet Epidemiol. 2014;1(1):2. 26401319 10.1186/2052-6687-1-2 PMC4574389

[50] Bagley RS . Common neurological disease of older animals. Vet Clin North Am Small Anim Pract. 1997;18:731‐742. 10.1016/s0195-5616(97)50134-3 9348638

[51] Conder GA , Sedlacek HS , Boucher JF , Clemence RG . Efficacy and safety of maropitant, a selective neurokinin 1 receptor antagonist, in 2 randomized clinical trials for prevention of vomiting due to motion sickness in dogs. J Vet Pharmacol Ther. 2008;31(6):528‐532. 19000275 10.1111/j.1365-2885.2008.00990.x

[52] Buckland EL , O'Neill D , Summers J , et al. Characterisation of antimicrobial usage in cats and dogs attending UK primary care companion animal veterinary practices. Vet Rec. 2016;179(19):489. 27543064 10.1136/vr.103830

[53] Who [Internet]. Critically Important Antimicrobials for Human Medicine, 5th revision. 2017 https://www.who.int/foodsafety/publications/antimicrobials-fifth/en/. Accessed October 16, 2019.

[54] Masters AK , Berger DJ , Ware WA , et al. Effects of short‐term anti‐inflammatory glucocorticoid treatment on clinicopathologic, echocardiographic, and hemodynamic variables in systemically healhty dogs. Am J Vet Res. 2018;79(4):411‐423. 29583045 10.2460/ajvr.79.4.411

[55] Tinklenberg RL , Murphy SD , Mochel JP , et al. Evaluation of dose‐response effects of short‐term oral prednisone administration on clinicopathologic and hemodynamic variables in healthy dogs. Am J Vet Res. 2020;81(4):317‐325. 32228253 10.2460/ajvr.81.4.317

